# Elevated plasma copeptin levels identify the presence and severity of non-alcoholic fatty liver disease in obesity

**DOI:** 10.1186/s12916-019-1319-4

**Published:** 2019-04-30

**Authors:** Ilaria Barchetta, Sofia Enhörning, Flavia Agata Cimini, Danila Capoccia, Caterina Chiappetta, Claudio Di Cristofano, Gianfranco Silecchia, Frida Leonetti, Olle Melander, Maria Gisella Cavallo

**Affiliations:** 1 Department of Experimental Medicine, Policlinico Umberto I, Sapienza University of Rome, Rome, Italy; 2 Department of Clinical Sciences, Lund University, Malmoe, Sweden; 3 Department of Medical-Surgical Sciences and Biotechnologies, Sapienza University of Rome, Rome, Italy

**Keywords:** Vasopressin, Copeptin, Antidiuretic hormone, Fatty liver, NAFLD, NASH, Metabolic syndrome, Obesity

## Abstract

**Introduction:**

Copeptin is the stable surrogate marker of vasopressin (VP), which is released in response to elevated plasma osmolality or low blood pressure. Elevated plasma copeptin levels are associated with higher risk of insulin resistance-related disorders, such as type 2 diabetes (T2DM), metabolic syndrome (MS), and cardiovascular disease, and experimental reduction of circulating VP levels is shown to significantly decrease hepatic fat content in obese rats, independently from body adiposity. However, the association between copeptin and non-alcoholic fatty liver disease and steatohepatitis (NAFLD/NASH) in humans has not been explored yet. The aim of this study was to explore the relationship between plasma copeptin and the presence/severity of NAFLD/NASH.

**Methods:**

For this study, we recruited 60 obese patients candidate to bariatric surgery for clinical purposes in which intraoperative liver biopsies were performed for diagnosing NAFLD/NASH. Circulating copeptin levels were also assessed in 60 age- and sex-comparable non-obese individuals without NAFLD at liver ultrasonography. Plasma copeptin was measured by sandwich immunoluminometric assay (Thermo Fisher Scientific).

**Results:**

Obese patients with biopsy-proven NAFLD (53%) had significantly higher copeptin levels than both obese individuals without NAFLD and non-obese subjects (ob/NAFLD+ 9.5 ± 4.9; ob/NAFLD− 6.4 ± 2.6; and non-ob/NAFLD− 7.4 ± 5.1 pmol/L; *p* = 0.004 and *p* = 0.01 respectively). Plasma copeptin concentration positively correlated with hepatic macro- and micro-vesicular steatosis (*r* = 0.36, *p* = 0.026; *r* = 0.31, *p* = 0.05), lobular inflammation (*r* = 0.37, *p* = 0.024) and significantly increased throughout degrees of NASH severity, as expressed as absence, borderline, and overt NASH at the liver biopsy (*r* = 0.35, *p* = 0.01). Greater circulating copeptin predicted the presence of NASH with OR = 1.73 (95% CI = 1.02–2.93) after multivariate adjustment for age, sex, renal function and presence of T2DM and MS components.

**Conclusions:**

Increased plasma copeptin is independently associated with the presence and severity of NAFLD and NASH, pointing to a novel mechanism behind human fatty liver disease potentially modifiable by pharmacological treatment and lifestyle intervention.

**Electronic supplementary material:**

The online version of this article (10.1186/s12916-019-1319-4) contains supplementary material, which is available to authorized users.

## Background

Vasopressin (VP) is a hormone secreted by the pituitary gland in response to increased plasma osmolality, low plasma volume, and low blood pressure. Copeptin is a cleavage product of the C-terminal part of the VP precursor which correlates well with plasma VP concentrations and which is easier to measure reliably [1, 2]. Thus, copeptin is nowadays considered the circulating surrogate biomarker of VP [2].

Beside its role in inducing reactive vasoconstriction and inhibiting diuresis and, thus, controlling blood volume [1, 2], VP exerts major effects on glucose and lipid metabolism by stimulating the hepatic glycogenolysis [3], gluconeogenesis [4], and fat production [5] and by modulating the release of insulin and glucagon from the pancreatic Langerhans’ islets [6].

In humans, elevated circulating copeptin levels have been independently associated with increased risk of type 2 diabetes mellitus (T2DM), cardiovascular morbidity and mortality [7–14], and clinical signatures of metabolic syndrome (MS), such as hyperinsulinemia [7], visceral fat deposition, systemic hypertension, high triglycerides, and impaired glucose regulation, independently from obesity [15, 16]. Of note, obese rats with elevated VP develop glucose intolerance, whereas blocking of the VP 1a receptors (V1aR) improves glucose tolerance [17].

Among metabolic disorders, non-alcoholic fatty liver disease (NAFLD) has gained special attention in the last decade since it represents the most common chronic liver disease worldwide with an estimated prevalence around 25% in the general population and up to 80% in obese individuals [18–20]. NAFLD is an established risk factor for all-cause and cardiovascular mortality [21]. However, mechanisms behind NAFLD development and progression to steatohepatitis (NASH) have not been fully unraveled, and no specific therapy has been identified yet [22].

VP exerts a direct influence on the hepatic fat metabolism by stimulating the production of triglycerides in rat hepatocytes [5]; accordingly, mice lacking expression of the VP receptor 1a have low triglycerides compared to wild type [23]. Indeed, obese rats with water-induced reduction of circulating VP levels have significantly decreased hepatic fat content compared with control obese rats, independently from changes in body adiposity [17]. In humans, clinical observations show a relationship between elevated plasma copeptin levels and the presence of hepatic cirrhosis, likely reflecting underlying circulatory dysfunction [24] suggesting a role of copeptin as a prognostic marker of liver disease [25].

Even though epidemiological and experimental evidence supports a direct involvement of VP in insulin resistance-associated disorders, to date nothing is known about the role of circulating copeptin in human NAFLD and NASH. Therefore, the aims of this study are to investigate the association between plasma copeptin and the presence and severity of NAFLD/NASH and to analyze clinical correlates of increased copeptin levels.

## Methods

### Study population

For this cross-sectional study, we recruited 60 consecutive obese subjects referring to our outpatient clinics at the Sapienza University of Rome, Italy, who were all candidates to bariatric surgery as for clinical indication, and an additional study cohort of 60 non-obese controls with comparable age and sex, who were not affected by metabolic syndrome (MS), as defined by the ATP III criteria [26], at the clinical evaluations performed in the same setting.

Eligible for this study were all the participants who had met the following inclusion criteria: male or female individuals aged between 20 and 65 years; no history of excessive alcohol drinking (considered as an average daily consumption of alcohol > 30 g/day in men and > 20 g/day in women); negative tests for the presence of hepatitis B surface antigen and antibody to hepatitis C virus; absence of history of cirrhosis and other causes of liver diseases (hemochromatosis, autoimmune hepatitis, Wilson’s disease); and no treatment with drugs known to cause liver steatosis (e.g., corticosteroids, estrogens, methotrexate, tetracycline, calcium channel blockers, or amiodarone). Additional exclusion criteria for subjects belonging to the control group were diagnosis of obesity defined as BMI ≥ 30 kg/m^2^, presence of MS [26], impaired glucose tolerance or T2DM, as defined by the American Diabetes Association 2009 criteria [27] and detection of hepatic steatosis at the abdomen ultrasonography assessment [28].

All the study participants underwent medical history collection, clinical work-up, and laboratory tests. Weight and height were measured with light clothes and without shoes, and the body mass index (BMI, kg/m^2^) was calculated. Waist circumference (cm) was measured midway between the 12th rib and the iliac crest. Systemic systolic (SBP) and diastolic (DBP) blood pressure were measured after 5 min resting; three measurements were taken, and the average of the second and third measurements was recorded and used in the analyses.

### Laboratory tests

Venous blood sampling after 12-h fasting was performed in all the study participants for routine analyses and metabolic characterization. Total cholesterol (mg/dL), high-density lipoprotein cholesterol (HDL, mg/dL), triglycerides (mg/dL), aspartate aminotransferase (AST, IU/L), alanine aminotransferase (ALT, IU/L), gamma-glutamyl transpeptidase (GGT, mg/dL), fasting blood glucose (FBG, mg/dL), uric acid (mg/dL), and creatinine (mg/dL) were measured by centralized standard methods. Fasting insulin (FBI, μΙU/mL) was measured by radioimmunoassay (ADVIA Insulin Ready Pack 100; Bayer Diagnostics, Milan, Italy), with intra- and inter-assay coefficients of variation < 5%, by an experienced lab technician, at Sapienza University, Rome, Italy. Low-density lipoprotein (LDL) cholesterol value was obtained using the Friedewald formula; the homeostasis model assessment of insulin resistance (HOMA-IR) and insulin secretion (HOMA-β%) was calculated as previously described [29].

Fasting copeptin was measured in plasma samples frozen immediately after separation and stored at − 80 °C for some weeks, using a KRYPTOR Compact Plus device and commercially available chemiluminescence sandwich immunoassay copeptin ProAVP kit with coated tubes (Thermo Scientific BRAHMS Copeptin proAVP KRYPTOR).

### Liver histology

Obese patients underwent intraoperative liver biopsy during surgery for sleeve gastrectomy; all the procedures were conducted in accordance with recommendations by the American Association for the Study of Liver Diseases [30]. A single pathologist blinded to patients’ medical history and biochemistry performed the overall histological evaluations. A minimum biopsy length of 15 mm or the presence of 10 complete portal tracts was required [31] for clinical diagnosis. Liver fragments were fixed in buffered formalin for 2–4 h and embedded in paraffin; sections were cut and stained with hematoxylin and eosin and Masson’s trichrome stains. Liver biopsy samples were classified based on the presence of NASH by Brunt definition [32] and graded according to the NAFLD activity score (NAS) [33]; fibrosis was quantified on the basis of the NASH Clinical Research Network Scoring System Definition [33].

### Liver ultrasound assessment

Study participants belonging to the control group underwent abdomen ultrasound (US) evaluation performed by the same operator blinded for patients’ identity and blood tests using an Esaote Medica instrument with a convex 3.5 MHz probe. The presence of fatty liver was defined based on the criteria by Saverymuttu et al. [28] which take into account abnormally intense, high-level echoes arising from the hepatic parenchyma, liver-kidney difference in echo amplitude, echo penetration into the deep portion of the liver, and clarity of liver blood vessel structure.

### Statistics

As far as we know, this is the first study investigating plasma copeptin levels in relation to the presence of NAFLD. Thus, for confirming the statistical power of our findings, we performed a post hoc sample size calculation on the basis of mean ± standard deviation (SD) copeptin levels in obese subjects with and without biopsy-proven NAFLD which showed that 27 subjects in each group would have been sufficient to reach the statistical significance with power = 80% and α error = 0.05.

All the statistic procedures have been performed using SPSS version 23. Values are shown as mean ± SD, median (interquartile range), or percentage, as appropriate. In this study population, plasma copeptin, triglycerides, FBG, AST, ALT, GGT, FBI, HOMA-IR, and HOMA-β% had skewed distribution and were log-transformed before the analyses. Mean values between two independent groups were compared by Student’s *T* test for continuous variables and by *χ*^2^ test for categorical parameters; comparisons between more than two subgroups were performed by the Bonferroni-adjusted ANOVA test. Bivariate correlations were explored by Pearson’s (continuous variables) or Spearman’s (categorical variables) coefficients. In order to identify determinants of NASH (yes/no, dependent variable) in our study population, we built multivariate logistic regression models including age, sex, T2DM, and components of MS, as expressed as either the numbers of MS components—ranging from 0 to 5—or entering each metabolic parameter as a continuous variable in a conditional forward logistic regression. Moreover, the predictive value of plasma copeptin for NASH identification was estimated by the area under receiver operating characteristic curve (AUROC), with a 95% confidence interval (CI).

A two-tailed *p* value < 0.05 was considered statistically significant, with a 95% confidence interval.

The study protocol was reviewed and approved by the local Ethics Committee at the Sapienza University of Rome, Italy, and conducted in conformance with the Helsinki Declaration. Informed written consent was obtained from all patients before all the study procedures.

## Results

Obese patients with biopsy-proven NAFLD (obese+/NAFLD+, 53%) had significantly higher copeptin levels than obese individuals without NAFLD (obese+/NAFLD−) (obese+/NAFLD+ 9.5 ± 4.9 pmol/L vs obese+/NAFLD− 6.4 ± 2.6 pmol/L, *p* = 0.004). Moreover, copeptin levels were also greater in obese+/NAFLD+ individuals when compared with non-obese subjects without MS and NAFLD (obese−/NAFLD−, mean ± SD copeptin 7.4 ± 5.1 pmol/L; *p* = 0.01).

The presence of significantly greater copeptin levels in obese+/NAFLD+ than both obese+/NAFLD− and obese−/NAFLD− was confirmed by the post hoc adjusted ANOVA test (model F = 4.09, *p* = 0.019; *p* = 0.022 and *p* = 0.034), whereas no statistically significant difference was observed between plasma copeptin in obese individuals without NAFLD and non-obese control subjects (Table 1).

**Table 1:** Clinical and biochemical characteristic of non-obese and obese study participants according to the presence of NAFLD

	Non-obese individuals (*n* = 60)	Obese no NAFLD (*n* = 28)	Obese NAFLD (*n* = 32)	*p* value ANOVA	*p* value Bonferroni post hoc test
Age (years)	46.3 ± 11.6	40.5 ± 12	43.2 ± 9.4	0.71	0.97^ 0.90* 0.70°
Sex (M/F)	60%	61.5%	32%	0.12^#^	0.14^^»^ 0.99*^»^ 0.59°^»^
BMI (kg/m^2^)	24.3 ± 3.2	43.5 ± 6.3	41.8 ± 4.3	< 0.001	0.20^ < 0.001^§^
Waist circumference (cm)	83.9 ± 13	129.4 ± 16.9	128 ± 7.7	< 0.001	0.94^ < 0.001^§^
SBP (mmHg)	115.5 ± 20.4	133 ± 8.4	124.4 ± 7.7	0.02	0.90^ 0.05* 0.06°
DBP (mmHg)	75.8 ± 8.1	85 ± 8.7	85.9 ± 22.1	0.009	0.98^ 0.10* 0.02°
Serum creatinine (mg/dL)	0.74 ± 0.15	0.70 ± 0.1	0.82 ± 0.16	0.03	0.04^ 0.68* 0.09°
Total cholesterol (mg/dL)	199 ± 35.1	213 ± 140.4	171 ± 126	0.18	0.16^ 0.28* 0.81°
HDL-C (mg/dL)	56.1 ± 14.1	52 ± 8.8	46.7 ± 10.2	0.037	0.82^ 0.46* 0.03°
LDL-C (mg/dL)	118.9 ± 34.8	141.9 ± 26.6	121.1 ± 22.3	0.35	0.33^ 0.39* 0.96°
Triglycerides (mg/dL)	77.5 (66–137.5)	101 (73.7–121.5)	136 (117.2–164)	0.09	0.59^ 0.14* 0.48°
Uric acid (mg/dL)	4.3 ± 1.2	4.9 ± 0.6	5.9 ± 1.5	0.02	0.02^ 0.58* 0.02°
AST (IU/L)	18 (15–21.25)	20.5 (17.8–25.3)	23 (17–30)	0.004	0.52^ 0.38* 0.003°
ALT (IU/L)	17 (13–24.25)	20.5 (15.8–27.3)	32 (18.5–47.5)	< 0.001	0.02^ 0.79* < 0.001°
FBG (mg/dL)	90.5 (82–95.25)	95 (92–101.5)	99 (87–125)	0.02	0.75^ 0.09* 0.02°
FBI (μΙU/mL)	10.3 (1.6–17.8)	9 (6.3–15.5)	11.5 (10.5–14)	0.66	0.85^ 0.91* 0.64
HOMA-IR	2.9 (2.1–4.8)	2 (1.5–3.6)	2.9 (2.4–3.8)	0.95	0.94^ 0.99* 0.94°
HOMA-β%	139.3 (101.2–336)	111.7 (68.1–180.4)	133.5 (67.4–216.7)	0.15	0.85^ 0.21* 0.26°
MS (%)	0%	46%	81%	< 0.001^#^	0.004^^»^ < 0.001^§»^
T2DM (%)	0%	8%	20%	0.01^#^	0.19^^»^ 0.04*^»^ 0.01°^»^
Treated with metformin		100%	70%		
Treated with glinides		0%	14%		
Treated with insulin		0%	28%		
Use of antihypertensive agents (%)	0%	36%	81%	< 0.001^#^	0.003^^»^ < 0.001^§»^
Use of statins (%)	0%	30%	69%	< 0.001^#^	0.003^^»^ < 0.001^§»^
Copeptin (pmol/L)	5.4 (4–9.45)	6.1 (4.3–8.6)	8.6 (5.4–11.9)	0.037	0.019^ 0.69* 0.022°

ANOVA test. ^#^Kruskall-Wallis test. Comparison between the following: ^§^non-obese individuals vs obese +/− NAFLD; ^obese NAFLD vs obese no NAFLD; *non-obese individuals vs obese no NAFLD; °non-obese individuals vs obese NAFLD. ^»^*χ*^2^ test for comparison between two groups. Data are expressed as a percentage, mean ± SD, and/or median (interquartile range), as appropriate

*Abbreviations*: *SBP* systolic blood pressure, *DBP* diastolic blood pressure, *HDL-C* high-density lipoprotein cholesterol, *LDL-C* low-density lipoprotein, *AST* aspartate aminotransferase, *ALT* alanine aminotransferase, *FBG* fasting blood glucose, *FBI* fasting blood insulin, *HOMA-IR* HOmeostasis Model Assessment of insulin resistance, *HOMA-β%* HOmeostasis Model Assessment of insulin secretion, *MS* metabolic syndrome, *T2DM* type 2 diabetes mellitus

When comparing plasma copeptin levels between obese+/NAFLD+ and obese+/NAFLD− patients in relation to the presence of MS, the finding of higher copeptin in presence of NAFL—both NAFLD and NASH —was confirmed in the MS group (*n* = 42; NAFLD− 7.1 ± 2.7 vs NAFLD+ 10 ± 5.2 pmol/L, *p* = 0.024; NASH− 7.7 ± 3.7 vs NASH+ 12.2 ± 5.7 pmol/L, *p* = 0.007), and slightly confirmed in the significantly smaller subgroup of obese patients without MS (*n* = 18; NAFLD− 5.9 ± 2.4 vs NAFLD+ 8 ± 2.4 pmol/L, *p* = 0.06; NASH− 6.5 ± 2.6 vs NASH+ 8.5 ± 0.2 pmol/L, *p* = 0.01). Furthermore, the association between higher copeptin levels and NAFLD persisted significantly when assessed in the partial correlation analysis adjusted for the presence of MS (correlation’s coefficient = 0.32, *p* = 0.017).

The presence of NAFLD correlated with higher copeptin levels and, as expected, with all the clinical parameters associated with MS, such as greater BMI, waist circumference, FBG, presence of T2DM, and atherogenic dyslipidemia, whereas no association was found between NAFLD, sex, and age (Additional file 1: Table S1).

**Fig. 1:**
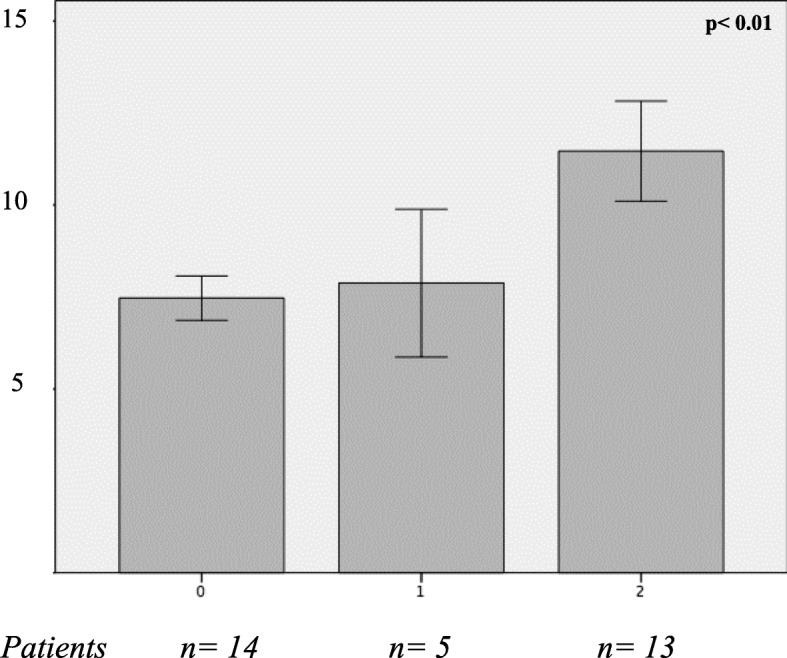
Plasma copeptin (pmol/L) in relation to the presence and severity of NASH (0 = absent; 1 = borderline; 2 = NASH). ANOVA test applied

In obese individuals, circulating copeptin levels positively correlated with the percentage of macro- and micro-vesicular steatosis, lobular inflammation, the NAS score for diagnosis of NASH, and the SAF score for fibrosis (Table 2). Furthermore, copeptin significantly increased throughout degrees of NASH severity, as expressed as absence (0, *n* = 14), borderline (1, *n* = 5), and overt (2, *n* = 13) NASH at the liver biopsy (Fig. 1).

**Table 2:** Copeptin-bivariate correlation analyses (Pearson’s coefficient, *Spearman’s coefficient, copeptin is considered as a continuous variable). Cohort obese individuals (*n* = 60)

	Correlation coefficient	*p* value
Age	0.048	0.75
Sex (M/F)	− 0.44	0.001*
BMI	0.03	0.84
Waist circumference	0.03	0.87
SBP (mmHg)	− 0.046	0.76
DBP	− 0.040	0.80
FBG	− 0.25	0.08
Total cholesterol	− 0.06	0.71
HDL	− 0.07	0.65
LDL	0.19	0.23
Triglycerides	− 0.21	0.18
AST	0.13	0.37
ALT	0.27	0.12
Serum creatinine	0.31	0.045
Serum uric acid	0.33	0.045
HOMA-β%	0.05	0.76
HOMA-IR	0.07	0.70
T2DM yes/no	− 0.20	0.15*
MS yes/no	− 0.16	0.29*
NAFL	0.31	0.015*
NASH	0.40	0.002*
NAS: macro-vesicular steatosis	0.36	0.026*
NAS: micro-vesicular steatosis	0.31	0.05*
NAS: lobular inflammation	0.37	0.024*
NAS score	0.35	0.03*
SAF score	0.44	0.001*

*Abbreviations*: *SBP* systolic blood pressure, *DBP* diastolic blood pressure, *HDL-C* high-density lipoprotein cholesterol, *LDL-C* low-density lipoprotein, *AST* aspartate aminotransferase, *ALT* alanine aminotransferase, *FBG* fasting blood glucose, *FBI* fasting blood insulin, *HOMA-IR* HOmeostasis Model Assessment of insulin resistance, *HOMA-β%* HOmeostasis Model Assessment of insulin secretion, *MS* metabolic syndrome, *T2DM* type 2 diabetes mellitus, *NAS* NAFLD activity score, *SAF* steatosis, activity, and fibrosis

Moreover, higher copeptin was associated with male sex and greater serum uric acid and creatinine concentration in all the study participants (Table 2 and Additional file 1: Table S2). In the entire study population, plasma copeptin concentration positively correlated also with greater waist circumference and with a higher prevalence of MS and NAFLD (Additional file 1: Table S2).

When comparing clinical characteristics of study participants in relation to the circulating copeptin levels, we observed that patients belonging to the highest copeptin quartile had a higher prevalence of male sex, MS, NAFLD and NASH and greater waist circumference, serum creatinine, and uric acid than those belonging to the lowest quartile (Table 3). Furthermore, higher copeptin concentration predicted the presence of NASH at the liver biopsy in the ROC curve with AUROC = 0.822 (95% CI 0.71–0.93, *p* < 0.001, Additional file 1: Figure S1).

**Table 3:** Clinical characteristics to study participants (*n* = 120) in relation to the quartile of plasma copeptin concentration

	Copeptin quartile 1	Copeptin quartile 2	Copeptin quartile 3	Copeptin quartile 4	*p* value
Age (years)	43.8 ± 9.2	46.2 ± 10.7	43.6 ± 12	45.7 ± 10.1	0.48
Sex (% M)	50%	30%	30%	90%	< 0.001*;0.01^
BMI (kg/m^2^)	32.6 ± 10.9	32.3 ± 9.8	36.9 ± 10.8	34.7 ± 11.2	0.51
Waist circumference(cm)	92.7 ± 19.3	100.5 ± 27.4	102.2 ± 27.12	113.4 ± 22.8	0.013
PAS (mmHg)	115 ± 29.4	125.4 ± 14.4	120.7 ± 13	124.4 ± 14.5	0.16
PAD (mmHg)	77.1 ± 6.3	85.5 ± 21.7	81.6 ± 9.25	80.5 ± 10.6	0.17
FBG (mg/dL)	91 (86.5–94.5)	94.5 (84.5–122)	94 (86.5–103.5)	95 (85.3–100.5)	0.56
FBI (μΙU/mL)	11.1 (10.6–20.3)	12.2 (8.7–25.2)	12.1 (8.8–27.4)	11.3 (8.8–17.2)	0.96
HOMA-IR	3.3 (2.4–4.02)	3.6 (1.9–5.6)	2.8 (1.97–5.9)	2.73 (1.8–4.3)	0.60
HOMA-β%	151.7 (122.2–496.1)	89.6 (63.8–121)	163.5 (107.9–274)	94 (133.5–199.7)	0.056
AST (IU/L)	20 (15–22.3)	20 (16–23)	23 (17.5–29)	18 (15.3–25.3)	0.62
ALT (IU/L)	21 (15.7–24.3)	18 (15–28)	25 (16.5–48)	18 (15–36.5)	0.36
GGT (IU/L)	16 (12.5–20)	13 (11–18.8)	25 (16.5–48)	18 (10.8–22.5)	0.53
Total cholesterol (mg/dL)	200.6 ± 49.6	205.9 ± 36	180.9 ± 27	204.5 ± 30.5	0.75
HDL (mg/dL)	55.4 ± 13.6	56.1 ± 14.4	43.6 ± 7.8	52.4 ± 11.5	0.44
LDL (mg/dL)	115.1 ± 35.2	123.3 ± 34	111.7 ± 25.4	122.7 ± 37.4	0.51
Triglycerides (mg/dL)	83 (72–139)	127 (73–164)	118.5 (87–146.5)	102.5 (73.5–150.3)	0.43
Serum creatinine (mg/mL)	0.68 ± 0.09	0.8 ± 0.15	0.74 ± 0.013	0.8 ± 0.17	0.01
Serum uric acid (mg/mL)	4.8 ± 1.02	5.5 ± 1.4	5.12 ± 1.1	6.1 ± 1.6	0.037
NAFLD (%)	15%	28%	40%	42%	0.026*; 0.09^
Biopsy-proven NASH	10%	0%	40%	50%	0.05*;0.004^
T2DM (%)	7%	15%	12%	0%	0.18*; 0.16^
MS (%)	26%	26%	48%	52%	0.051*; 0.22^

*Chi-square test. *p* values related to the comparison between copeptin quartiles 1 vs 4. ^*p* value related to distribution across all the quartiles (Kruskal-Wallis test)

*Abbreviations*: *BMI* body mass index, *SBP* systolic blood pressure, *DBP* diastolic blood pressure, *HDL-C* high-density lipoprotein cholesterol, *LDL-C* low-density lipoprotein, *AST* aspartate aminotransferase, *ALT* alanine aminotransferase, *GGT* gamma-glutamyl transpeptidase, *FBG* fasting blood glucose, *FBI* fasting blood insulin, *HOMA-IR* HOmeostasis Model Assessment of insulin resistance, *HOMA-β%* HOmeostasis Model Assessment of insulin secretion, *NAFLD* non-alcoholic fatty liver disease, *NASH* non-alcoholic steatohepatitis, *MS* metabolic syndrome, *T2DM* type 2 diabetes mellitus

Finally, in a logistic regression model adjusted for age, sex, renal function, presence of T2DM, and MS components, copeptin levels predicted the presence of NASH at the liver biopsy (Table 4). The association between copeptin and NASH persisted statistically significant also after further adjustment for each individual metabolic parameter (BMI, FBG, triglycerides and HDL-c), entered as continuous variables in progressive conditional forward regression models (copeptin-standardized β-coefficient = 0.64, *p* = 0.035, odds ratio = 1.97, 95% CI = 1.05–3.69; Cox and Snell *R*^2^ = 0.56; Additional file 1: Table S3).

**Table 4:** Multivariate logistic regression analysis. The presence of NASH is the dependent variable. Copeptin is considered as a continuous variable

	*β*	S.E.	Wald	β-standardized	*p* value	Odds ratio	95% CI	
							Lower	Upper
Age	0.33	0.17	3.47	0.63	0.06	1.39	0.98	1.95
Sex (M/F)	− 1.08	1.88	0.33	− 0.11	0.57	0.34	0.008	13.57
Copeptin	0.547	0.27	4.09	0.54	0.043	1.73	1.02	2.93
T2DM (yes/no)	1.65	1.85	0.79	0.13	0.37	5.2	0.14	197.5
Serum creatinine	0.57	5.4	0.01	− 0.02	0.92	1.77	0.000	7420
Dyslipidemia (yes/no)	0.43	2.80	2.41	0.03	0.12	0.013	0.000	3.12
Number of MS components	3.46	1.71	4.09	0.36	0.043	31.8	1.11	909.1

Cox and Snell *R*^2^ = 0.408

*Abbreviations*: *S.E.* standard error, *CI* confidence interval, *T2DM* type 2 diabetes mellitus, *MS* metabolic syndrome

## Discussion

In this study, we demonstrated for the first time in humans the existence of a strong association between elevated circulating copeptin levels and the presence of NAFLD and NASH. Higher plasma copeptin was associated with greater intrahepatic fat content and inflammation at the liver histology as well as increasing severity of NASH.

We also show an association between copeptin, visceral obesity, and the presence of MS, a finding in line with previous studies demonstrating a role of copeptin in glucose and lipid metabolism [3, 4, 6, 17, 34] which likely underlies the increased risk of metabolic and cardiovascular diseases associated to elevated copeptin observed in many longitudinal studies [7–16].

The predictive value of copeptin in identifying NAFLD and NASH in obese individuals was independent from other dysmetabolic conditions, such as increased body adiposity and the diagnosis of T2DM and MS, even if an association between higher copeptin and metabolic impairment was also observed. In our population of obese individuals, the prevalence of MS reached 70% whereas T2DM was diagnosed in 14% and biopsy-proven NAFLD in just over 50% participants. Therefore, we may speculate that, according to the metabolic profiling, at least one third of these patients had not (yet) clinically relevant metabolic impairment but obesity, at the time of study recruitment, thus explaining the observation of some overlap in few parameters, such as fasting insulin and its derived indexes, between obese and non-obese subjects.

In line with previous investigations, we observed an association between male sex and greater copeptin concentration. In order to exclude the possible interference of sex distribution behind the association between copeptin and NAFLD, we first explored the association between NAFLD and sex, finding no relationship between these two variables. Thereafter, we built several sex- and age-forced multivariate models confirming that sex did not represent a confounder in the association between NAFLD and higher copeptin levels. Furthermore, in our study, plasma copeptin and histological parameters associated with NASH correlated in a dose-dependent manner, which may represent a direct action of VP on liver parenchyma.

The only previous investigation on VP and liver steatosis has been conducted on animal models by Taveau and collaborators [17], who explored the impact of experimentally induced high and low circulating VP levels on glucose homeostasis in obese rats. The authors demonstrated impaired glucose regulation in the presence of high VP levels and a surprisingly reduced hepatic fat content after lowering circulating VP concentration by exposing obese rats to 2-week high water intake. As compared with control obese rats, water-induced low VP reduced the intrahepatic lipid content and the expression of proteins involved in lipogenesis whereas it increased the hepatic glycogen content [17]. Intriguingly, the authors observed an unexpected dissociation between the effects of decreased VP levels on glucose homeostasis and hepatic fat accumulation: low-VP rats did not display a significantly improved glucose tolerance even though they exhibited a marked decrease in hepatic steatosis, whereas rats treated with VP infusion in the same study exhibited impaired glucose tolerance but no aggravation of hepatic steatosis [17].

Liver is a well-known target of VP action, and the V1aR is widely expressed in hepatic tissue, where it mainly regulates lipid metabolism promoting lipogenesis [23, 35] and stimulating bile acid production [23]; accordingly, V1aR knock-out mice have low blood triglycerides and increased ketone bodies when compared to wild type, confirming the antilipolytic action of VP [23]. VP/V1aR system has been widely studied in relation to the progression to cirrhosis and hepatic decompensation of several acute and chronic liver diseases [24, 25, 36–38]. Indeed, the secretion of VP is involved in advanced stages of chronic liver diseases; VP counteracts the reduction of the arterial pressure induced by splanchnic vasodilatation and subsequent increased peripheral vascular resistance. In this scenario, although the non-osmotic secretion of VP may temporarily preserve the arterial blood volume, it is associated with the development of ascites, hepato-renal syndrome, and detrimental clinical outcomes. Thus, Solà and collaborators [24] first demonstrated that plasma copeptin represents a prognostic factor for disease progression, clinical decompensation, and prognosis in patients with cirrhosis [24], and these findings were confirmed in other investigations [36–38]. In our study, no participant had any clinical or biochemical signs of hepatic damage, making it unlikely that mechanisms involving fluid distribution and vascular compliance underlie the association between high copeptin and NAFLD in our study.

The cross-sectional design of our study does not allow us to establish a causal nexus between these findings; however, it is plausible to hypothesize that increased VP, as measured by plasma copeptin levels, play a direct role in inducing intrahepatic fat accumulation by modulating lipid metabolism towards antilipolysis. The VP system has been hypothesized to represent a unifying factor behind the MS and a link between metabolic and cardiovascular disease [9, 16]. As liver steatosis is considered as a feature of the MS, our findings add further knowledge on metabolic complications associated with an impaired VP/V1aR system and provide novel insights on possible mechanisms underlying NAFLD and NASH.

## Conclusion

Our study demonstrates for the first time that copeptin levels predict the presence of biopsy-proven NAFLD in obese individuals, regardless of body adiposity and the coexistence of other metabolic disorders. Studies with longitudinal design are warranted for exploring the possible involvement of AVP/V1aR system in the development and progression of NAFLD.

## Additional file

Additional file 1: **Table S1.** Presence of NAFLD - Bivariate correlation analyses. Spearman’s coefficient, NAFLD is considered as a dichotomous variable (Yes/No). **Table S2.** Copeptin- Bivariate correlation analyses (Pearson’s coefficient, *Spearman’s coefficient, copeptin is considered as a continuous variable). **Table S3.** Multivariate logistic regression analysis. The presence of NASH is the dependent variable. Copeptin is considered as a continuous variable. **Figure S1.** Copeptin area under the receiver operating characteristic (ROC) curve for NASH. (DOCX 77 kb)
