# Supplementary material for: Elevated plasma copeptin levels identify the presence and severity of non-alcoholic fatty liver disease in obesity
Source: BMC Med. 2019 Apr 30;17:85. doi: 10.1186/s12916-019-1319-4 (PMC6489227; doi:10.1186/s12916-019-1319-4)
Supplement: Supplementary file 1 — Table S1. Presence of NAFLD - Bivariate correlation analyses. Spearman’s coefficient, NAFLD is considered as a dichotomous variable (Yes/No). Table S2. Copeptin- Bivariate correlation analyses (Pearson’s coefficient, *Spearman’s coefficient, copeptin is considered as a continuous variable). Table S3. Multivariate logistic regression analysis. The presence of NASH is the dependent variable. Copeptin is considered as a continuous variable. Figure S1. Copeptin area under the receiver operating characteristic (ROC) curve for NASH. (DOCX 77 kb) [file 12916_2019_1319_MOESM1_ESM.docx]

**Additional file 1**

**Entire population (n= 120)**

|  | *Correlation coefficient* | *p-value* |
| --- | --- | --- |
| Age | -0.05 | 0.62 |
| Sex (M/F) | -0.01 | 0.86 |
| BMI | 0.52 | <0.001 |
| Waist circumference | 0.66 | <0.001 |
| SBP | 0.18 | 0.11 |
| DBP | 0.22 | 0.05 |
| Copeptin | 0.25 | 0.01 |
| FBG | 0.26 | 0.02 |
| Total Cholesterol | -0.09 | 0.40 |
| HDL | -0.27 | 0.02 |
| LDL | -0.06 | 0.59 |
| Triglycerides | 0.34 | 0.02 |
| AST | 0.23 | 0.04 |
| ALT | 0.39 | <0.001 |
| FBI | 0.04 | 0.78 |
| HOMA- β% | -0.08 | 0.59 |
| HOMA-IR | 0.13 | 0.37 |
| T2DM yes/no | 0.31 | 0.002 |
| MS yes/no | 0.64 | <0.001 |
| Number of MS’ components | 0.68 | <0.001 |

**Table S1.** Presence of NAFLD - Bivariate correlation analyses. Spearman’s coefficient, NAFLD is considered as a dichotomous variable (Yes/No)

*Abbreviations: BMI – Body Mass Index, SBP – Systolic Blood Pressure, DBP – Diastolic Blood Pressure, FBG - fasting blood glucose, HDL-C - High-Density Lipoprotein Cholesterol, LDL-C - Low-Density Lipoprotein, AST - aspartate aminotransferase, ALT - alanine aminotransferase, GGT - gamma-glutamyl transpeptidase, FBI - Fasting blood insulin, HOMA-IR - HOmeostasis Model Assessment of insulin resistance, HOMA-β% - HOmeostasis Model Assessment of insulin secretion, MS – Metabolic Syndrome, T2DM – Type 2 Diabetes Mellitus.*

**Entire population (n= 120)**

|  | *Correlation coefficient* | *p-value* |
| --- | --- | --- |
| Age | 0.089 | 0.38 |
| Sex (M/F) | -0.34 | 0.001* |
| BMI | 0.06 | 0.53 |
| Waist circumference | 0.26 | 0.034 |
| SBP | 0.09 | 0.39 |
| DBP | 0.02 | 0.86 |
| FBG | -0.02 | 0.80 |
| Total Cholesterol | -0.008 | 0.94 |
| HDL-c | -0.14 | 0.18 |
| LDL-c | -0.08 | 0.48 |
| Triglycerides | -0.07 | 0.50 |
| AST | 0.10 | 0.32 |
| ALT | 0.18 | 0.08 |
| GGT | 0.20 | 0.15 |
| Serum creatinine | 0.26 | 0.012 |
| Serum uric acid | 0.37 | 0.015 |
| NAFLD (yes/no) | 0.25 | 0.01 |
| T2DM (yes/no) | -0.13 | 0.17* |
| MS (yes/no) | 0.23 | 0.014* |

**Table S2.** Copeptin- Bivariate correlation analyses (Pearson’s coefficient, *Spearman’s coefficient, copeptin is considered as a continue variable)

*Abbreviations: BMI – Body Mass Index, SBP – Systolic Blood Pressure, DBP – Diastolic Blood Pressure, HDL-C - High-Density Lipoprotein Cholesterol, LDL-C - Low-Density Lipoprotein, , FBG - fasting blood glucose, AST - aspartate aminotransferase, ALT - alanine aminotransferase, GGT - gamma-glutamyl transpeptidase, FBG - fasting blood glucose, NAFLD – non-alcoholic fatty liver disease, NASH – non-alcoholic steatohepatitis, MS – Metabolic Syndrome, T2DM – Type 2 Diabetes Mellitus.*

|  |  | β | S.E. | Wald | β standardized | P value | Odd Ratio | 95% C.I.  Lower Upper | |
| --- | --- | --- | --- | --- | --- | --- | --- | --- | --- |
| Model 1^a^ | Sex | 2.724 | 2.216 | 1.510 | 0.27 | 0.219 | 15.239 | 0.198 | 1173.787 |
|  | Age | 0.144 | 0.087 | 2.773 | 0.30 | 0.096 | 1.155 | 0.975 | 1.370 |
|  | BMI | 0.003 | 0.162 | 0.000 | 0.004 | 0.986 | 1.003 | 0.731 | 1.376 |
|  | **Copeptin** | **0.677** | **0.321** | **4.450** | **0.64** | **0.035** | **1.968** | **1.049** | **3.691** |
|  | HDL-c | -0.201 | 0.109 | 3.377 | -0.45 | 0.066 | 0.818 | 0.661 | 1.013 |
|  | Triglycerides | 0.036 | 0.019 | 3.592 | 0.75 | 0.058 | 1.037 | 0.999 | 1.076 |
|  | FBG | -0.047 | 0.053 | 0.785 | 0.39 | 0.375 | 0.954 | 0.860 | 1.058 |
|  | Constant | -6.475 | 8.969 | 0.521 |  | 0.470 | 0.002 |  |  |
| Model 2^a^ | Sex | 2.729 | 2.198 | 1.542 | 0.27 | 0.214 | 15.317 | 0.206 | 1137.337 |
|  | Age | 0.145 | 0.087 | 2.781 | 0.31 | 0.095 | 1.155 | 0.975 | 1.369 |
|  | **Copeptin** | **0.676** | **0.320** | **4.471** | **0.64** | **0.034** | **1.967** | **1.051** | **3.682** |
|  | HDL-c | -0.200 | 0.107 | 3.530 | -0.45 | 0.060 | 0.819 | 0.664 | 1.009 |
|  | Triglycerides | 0.036 | 0.018 | 3.933 | 0.75 | 0.047 | 1.037 | 1.000 | 1.074 |
|  | FBG | -0.047 | 0.053 | 0.797 | 0.39 | 0.372 | 0.954 | 0.861 | 1.058 |
|  | Constant | -6.352 | 5.733 | 1.228 |  | 0.268 | 0.002 |  |  |
| Model 3^a^ | Sex | 2.819 | 2.202 | 1.639 | 0.27 | 0.200 | 16.767 | 0.224 | 1256.396 |
|  | Age | 0.104 | 0.071 | 2.132 | 0.22 | 0.144 | 1.110 | 0.965 | 1.276 |
|  | **Copeptin** | **0.634** | **0.295** | **4.627** | **0.60** | **0.031** | **1.884** | **1.058** | **3.357** |
|  | HDL-c | -0.192 | 0.102 | 3.501 | -0.43 | 0.061 | 0.826 | 0.676 | 1.009 |
|  | Triglycerides | 0.032 | 0.017 | 3.626 | 0.67 | 0.057 | 1.033 | 0.999 | 1.067 |
|  | Constant | -8.612 | 5.429 | 2.516 |  | 0.113 | 0.000 |  |  |
| Model 4^a^ | Age | 0.093 | 0.069 | 1.809 | 0.20 | 0.179 | 1.098 | 0.958 | 1.258 |
|  | **Copeptin** | **0.353** | **0.146** | **5.852** | **0.37** | **0.016** | **1.423** | **1.069** | **1.894** |
|  | HDL-c | -0.130 | 0.082 | 2.473 | -0.30 | 0.116 | 0.878 | 0.747 | 1.032 |
|  | Triglycerides | 0.025 | 0.014 | 3.248 | 0.58 | 0.071 | 1.026 | 0.998 | 1.054 |
|  | Constant | -5.432 | 4.224 | 1.654 |  | 0.198 | 0.004 |  |  |
| Model 5^a^ | **Copeptin** | **0.323** | **0.140** | **5.375** | **0.32** | **0.020** | **1.382** | **1.051** | **1.817** |
|  | HDL-c | -0.084 | 0.054 | 2.489 | -0.18 | 0.115 | 0.919 | 0.827 | 1.021 |
|  | Triglycerides | 0.028 | 0.014 | 4.172 | 0.61 | 0.041 | 1.028 | 1.001 | 1.056 |
|  | Constant | -3.477 | 3.288 | 1.118 |  | 0.290 | 0.031 |  |  |

^a^ Variables entered on Model 1: Sex, Age, BMI, Copeptin, HDL-c, Triglycerides, FBG.

Cox & Snell R^2^ = 0.56

**Table S3.** Multivariate logistic regression analysis. The presence of NASH is the dependent variable. Copeptin is considered as a continuous variable.

*Abbreviations: S.E. - Standard Error, C.I. - Confidence Interval, BMI – Body Mass Index, HDL-c - High-Density Lipoprotein Cholesterol, FBG - fasting blood glucose*


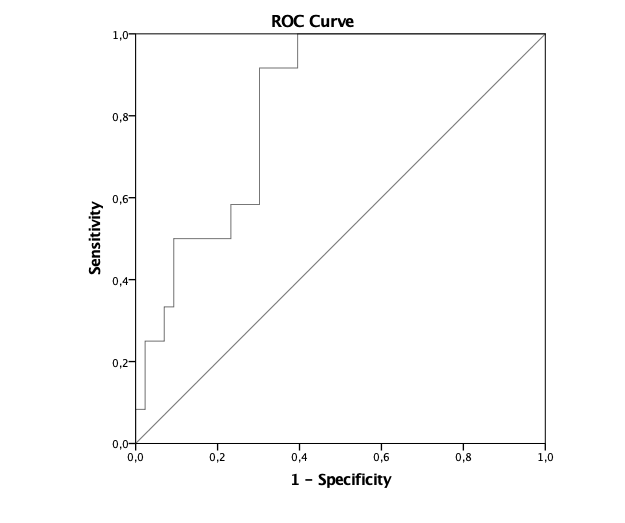


| Area | Std. Error^a^ | Asymptotic Sig.^b^ | Asymptotic 95% Confidence Interval | |
| --- | --- | --- | --- | --- |
|  |  |  | Lower Bound | Upper Bound |
| 0.822 | 0.057 | 0.001 | 0.709 | 0.934 |
| a. Under the nonparametric assumption | | | | |
| b. Null hypothesis: true area = 0.5 | | | | |

Sensitivity: 92% - Specificity: 70% per plasma copeptin values > 8.06 pmol/L

**Figure S1.** Copeptin area under the receiver operating characteristic (ROC) curve for NASH.
